# Classical Density Functional Treatment of Polydisperse Polarizable Clusters

**DOI:** 10.1021/acs.jpcb.5c08060

**Published:** 2026-03-23

**Authors:** Clifford E. Woodward, David Ribar, Jan Forsman

**Affiliations:** † School of Physical, Environmental and Mathematical Sciences University College, University of New South Wales, ADFA, Canberra ACT 2600, Australia; ‡ Computational Chemistry, 5193 Lund University, P.O.Box 124, S-221 00 Lund, Sweden

## Abstract

Ion clustering has been proposed as a mechanism leading to the peculiar “anomalous underscreening” phenomenon seen for electrostatic interactions between charged surfaces immersed in concentrated electrolytes. These interactions have been measured using the Surface Force Apparatus, according to which there are strong repulsive interactions between like-charged surfaces, with a range that increases upon further addition of salt, above some threshold concentration. A common suggestion is that ionic aggregates, if they form in sufficient numbers, will reduce the concentration of free ions and thereby increase the nominal Debye length. In previous work, we investigated a cluster model using classical Density Functional Theory (cDFT) and a polymer-like description of the ion clusters. These clusters were monodisperse and of either a linear or branched architecture, and a fixed charge sequence along the chains. In this work, we generalize the cDFT to treat “living polymers” with variable chain lengths and charge arrangements along the chain. This approach allows clusters to become polarized by the presence of charged surfaces, manifested by like-charged bonding. We find that even with a small degree of like-charged bonding a full equilibrium treatment of our model predicts only weak repulsion between like-charged surfaces. When a global constraint is applied so that the charged surfaces are neutralized only by the dissociated ions, while the clusters contribute overall zero charge, even a very small fraction of clustering ions generate strong and long-ranged forces. Moreover, if the cluster fraction increase substantially upon the addition of further salt, then the strength of the surface forces will also increase, although the range remains roughly constant.

## Graphical abstract

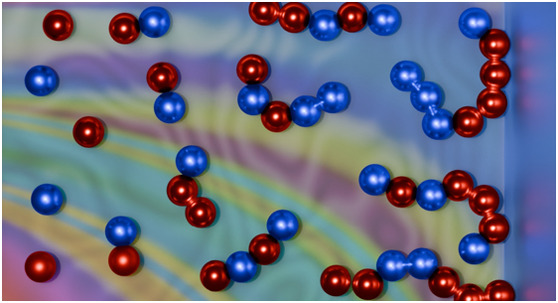

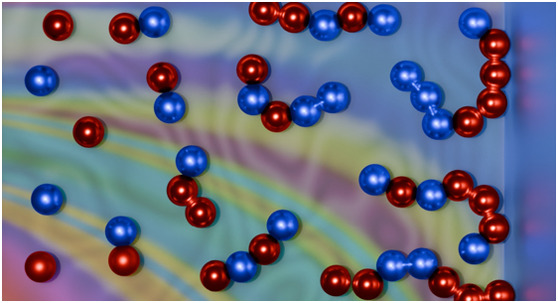

## Introduction

Interactions between charged surfaces immersed in electrolyte solutions have been the subject of extensive study for decades, owing to their fundamental importance in numerous biological and industrial systems. A widely used theoretical framework is based on the primitive model, where the solvent is treated as a dielectric continuum, and ion distributions are described within the mean-field Poisson–Boltzmann (PB) theory. For instance, this model forms the basis of the seminal DLVO theory, describing colloidal interactions.^1–3^

Extensions to the PB approach that account for ion–ion correlations are essential for understanding systems characterized by strong electrostatic coupling. Related phenomena such as charge inversion and like-charge attraction are now relatively well-understood. Within mean-field treatments of primitive models, the characteristic length scale governing electrostatic screening is the Debye length, λ_*D*_. This length quantifies the effective range of electrostatic interactions between charged surfaces in dilute electrolytes and decreases with increasing ionic concentration and/or charge, reflecting enhanced screening. However, experimental observations, primarily using the Surface Force Apparatus (SFA), have revealed striking deviations from this classical behavior.^4–8^ These studies report that the decay length of surface interactions in simple aqueous electrolytes and in ionic liquids can substantially exceed λ_*D*_, and, intriguingly, may increase with salt concentration beyond a certain threshold concentration. This counterintuitive result is often referred to as *anomalous underscreening*.^9^

Independent experimental evidence for anomalous underscreening has been obtained using techniques other than SFA. For example, Gaddam and Ducker^10^ employed fluorescence imaging to probe the spatial distribution of a negatively charged fluorescent tracer (at micromolar concentration) between charged silica surfaces. Their measurements revealed concentration-dependent decay lengths consistent with the anomalous trends seen in SFA experiments. Such an inverse relationship between screening length and ionic strength would also be expected to influence colloidal stability. Indeed, Yuan et al.^11^ reported re-entrant dispersion behavior in charged colloids, where aggregation at intermediate salt concentrations was followed by redispersion at very high concentrations.

It should also be noted that Kumar et al.^12^ employed atomic force microscopy (AFM) to study interactions between charged surfaces in aqueous electrolytes, but did not observe anomalous underscreening; at high ionic strengths, the measured forces were short-ranged. Baimpos et al. investigated *LiCl*_(*aq*)_ and *CsCl*_(*aq*)_ solutions using both SFA and AFM, finding long-range forces with the former but not the latter technique. They suggested that SFA measurements more accurately capture equilibrium interactions, whereas AFM may be limited by a lower sensitivity.

Although anomalous underscreening has now been reported by several independent groups, its physical origin remains unresolved.^8,9,13–21^ Existing theoretical models do not yet provide a convincing explanation. A commonly proposed mechanism involves ionic clustering,^4,5,9,21–24^ which is expected to become increasingly significant at high concentrations and strong coupling. In this view, the observed screening length reflects a renormalized Debye length determined primarily by a reduced population of “free” ions, since ion clusters are likely to carry little or no net charge.

Experimental evidence for ion clustering at high ionic strengths has also been reported.^25–32^ For example, concentrated *KCl*_(*aq*)_ and *NaCl*_(*aq*)_ solutions exhibit fluorescence,^33^ which Villa et al. have attributed to a stiffening of hydrogen-bond networks in hydration shells, thereby suppressing nonradiative decay pathways. Their interpretation was supported by quantum chemical calculations, suggesting that the fluorescing structures correspond to hydrated ion clusters.

However, ion clustering may in principle affect surface interactions in more ways than merely via the concomitant drop of the ionic strength. A recent study utilizing classical polymer Density Functional Theory, cDFT, suggested that solutions in which *all* ions are clustered, and treated as polymers, mediate remarkably strong and long-ranged repulsive forces between charged surfaces.^23^ There are a few noteworthy limitations to the model used in that work:

- the polymers formed (“ion clusters”) were overall monovalent and monodisperse
- the polymers had a fixed sequence of charges (in most cases alternating)
- there were no dissociated ions (simple salt) present

The model used linear polymers to model the clusters. This might also have seemed crude, but essentially identical results were established with corresponding models utilizing branched architectures. This implies that the linear sequence constitutes a reasonable model (note that even linear polymers will typically have a rather globular shape in solution).

In this work, we will generalize the cDFT to handle mixtures containing two different kinds of monomers (*A* and *B*, say), which in turn can polymerize to form *living* (or *equilibrium*) polymers, by forming reversible bonds (*A*–*A*, *A*–*B* and *B*–*B*), which produce a range of chain lengths *and* compositions. Moreover, both of these will respond to changes of the external conditions. For instance, the presence of a surface with a strong affinity to *A* will induce the formation of longer *A*-rich regions with the adjacent polymers. As an application to the electrolyte systems discussed above, the monomers (*A* and *B*) will refer to anions and cations specifically. The formation of like-charged bonded monomers in the chains (*A*–*A* and *B*–*B*) while energetically unfavorable (due to the Coulomb repulsion) will occur as a response to the application of external potentials due to, e.g., charged surfaces. If such bonding was disallowed then chains would be restricted to a sequence of alternating charges, which reduces the ability of our cluster model to manifest charge asymmetry or become charged in general. Below, we will use the term “polarization” to describe both these types of perturbation (asymmetry and charging) of the clusters. In the next section, we describe the cDFT in more detail.

## Model and Theory

The cDFT formulation presented here is in principle able to treat general *A*/*B* mixtures of monomers that form linear polymers with “dissociable bonds” of type *A*–*A*, *A*–*B* and *B*–*B*. However, in this application we are mainly concerned with the case where “*A*” is a monovalent cation (say) and “*B*” is a monovalent anion, both of which are dissolved in an implicit aqueous medium. There are special aspects to consider for such systems, compared with neutral monomers *A* and *B*. For instance, their mutual Coulomb interaction is expected to render *A*–*B* bonds stronger than *A*–*A* and *B*–*B*. We note that like-charged bonding may be advantageous in the presence of strongly charged surfaces of a valence which is opposite to that of the bonding monomers. The mutual repulsion of like-charged monomers is counteracted by the added attraction to the surface. Ion correlation effects can also enhance the presence of like-charged bonds as well as hydration forces.

The general picture is as follows. A local concentration fluctuation of ions may form large “bonded” clusters resembling a loose crystal, which traps some water molecules and displaces others. As already mentioned the degree of cluster polarization induced by the presence of charged surfaces is reflected in the degree of like-charged monomer bonding in our model. The presence of localized regions of like-charge in the cluster is also further facilitated by rearrangements of nearby ions and water molecules. For this reason, in our model, we let the degree of like-charged and unlike-charged bonding in the linear chain to be determined by variable parameters.

### Density Functional Theory of Linear Polarizable ion Clusters

We begin with the exact canonical free energy density functional for an ideal, monodisperse polymer fluid of flexible *r*-mers, which can consist of different types of monomers,

1 $$\beta F_{r}^{id}=\int dRN_{c}\left(R\right)\left(\ln\left[N_{c}\left(R\right)\right]-1\right)+\int dRN_{c}\left(R\right)\Phi^{\left(b\right)}\left(R\right)+\int \sum_{i}drn_{i}\left(r\right)\psi_{i}^{0}\left(r\right)$$

The vector subscript, **c** = (*r*, **s**), denotes both the degree of polymerization *r* (chain length), and the sequence of monomer types, **s** = (*s*_1_, *s*_2_,···*s*_*r*_), where the number of different monomer types may be different to *r*. In fact we now particularise to the case of only two different monomer types constituting “+” and “–” charges, so *s*_*i*_ = ⊕, ⊖.

As usual, β = 1/(*k*_*B*_ *T*), denotes the inverse thermal energy. The *r*-dimensional density, *N*_**c**_(**R**), is a function of the monomer configuration **R** = (**r**_1_,···,**r**_*r*_), where **r**_*k*_ is the coordinate of monomer *k* in the chain of type *s*_*k*_ and Φ^(*b*)^ (**R**) describes the bonds between connected monomers

2 $$\Phi^{\left(b\right)}\left(R\right)=\sum_{i=1}^{r-1}\phi^{\left(b\right)}\left(|r_{i}-r_{i+1}|\right)$$

We shall assume that the bonds have a fixed length *b*, but with full rotational freedom, i.e., there are no bond angle potentials. Finally, ψ_*i*_^(0)^(**r**), is the external potential acting on the monomer density, *n*_*i*_(**r**), of type *i* = +, −. The bulk fluid is assumed to contain equal numbers of “+” and “-” monomers in total, each with density *n*_*b*_.

Clusters are formed via a stepwise equilibrium growth, including reactions of the following type 
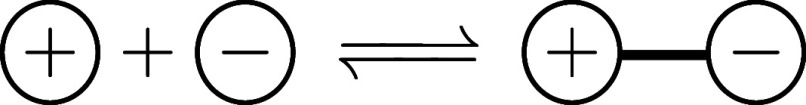
with a Gibbs reaction free energy change Δ*G*

$$\beta \Delta G=\beta \Delta G_{0}\left(+-\right)-l_{B}/b\equiv \kappa_{+-}$$

where *l*_*B*_ is the Bjerrum length, having a fixed value of 7.16 Å in this work. One could consider an alternative approximation where the last term in this equation includes ionic screening (screened Coulomb) but this is unimportant to the mechanisms discussed in this work. The formed ion pair can participate in a further reaction, for instance with another cation. There are then two options:
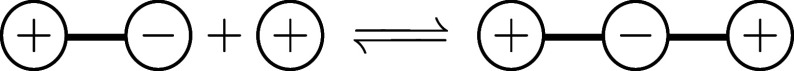
with a reaction free energy change

$$\beta \Delta G=\kappa_{+-}$$

or
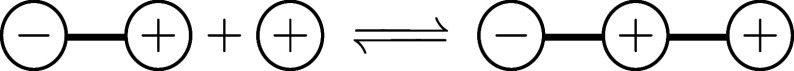
with a reaction free energy change

$$\beta \Delta G=\beta \Delta G_{0}\left(++\right)+l_{B}/b\equiv \kappa_{++}$$

We will assume that Δ*G*_0_(++) = Δ*G*_0_(−−), but Δ*G*_0_(++) may differ from Δ*G*_0_(+−), since these depend on short-ranged solvent-induced potential of mean forces, which in turn rely on molecular details at a level not explicitly accounted for in this work. Similar assumptions were made in recent simulation work, with various specific choices of the short-ranged solvent-induced potential of mean force (beyond dielectric screening) being investigated.^24^ A monomer can be bonded to either two (central monomers) or one (end monomers) nearest-neighbor (NN) monomers. We note that Bjerrum’s classical theory of ion association^34^ corresponds to a truncation of this polymerization process at the dimer level, restricted to electroneutral pairs. Our model thus constitutes a natural generalization of Bjerrum’s framework, permitting chain growth beyond dimers, polydispersity in both length and composition, and the formation of charged clusters. If the average density of aggregates is, ϕ_*p*_, and ⟨*r*⟩ is the average association number, then ϕ_*p*_⟨*r*⟩ = 2*n*_*b*_. The specific fractional distributions of polymer aggregate types is given by some function *F*(**c**). This distribution gives the fraction of species with internal structure **c** and is normalized according to

3 $$\sum_{c}F\left(c\right)=1$$

The ideal chemical potential of the various polymer species, **c**, in the bulk is then given by,

4 $$\beta \mu_{c}^{\left(\mathrm{id}\right)}=\ln\left[\phi_{p}F\left(c\right)\right]$$

As described above, *F*(**c**) restricts polymers to consist of simple linear chains of “+” and “–” monomers. The chains have variable length, and variable charge distributions, as determined by the bond parameters κ_++_ and κ_+–_.

In the presence of additional (nonbonding) interactions, the ideal functional must be supplemented with a suitable excess term, *F* ^*ex*^. Thus, the total grand potential functional is given by the following general expression

5 $$\Omega =F^{id}\left[\{N_{r}^{\left(c\right)}\left(R\right)\}\right]+F^{ex}\left[\{n_{i}\left(r\right)\}\right]-\sum_{r,c}\mu_{r,c}\int dRN_{r}^{\left(c\right)}\left(R\right)$$

where *r* represents the length of chain and *c* is the specific distribution of “+” and “–” monomers. More specifically the chains will consist of alternating segments of like charges. In the bulk fluid the average length of positive and negative segments will be equal, but this will generally not be the case in the presence of an applied electrostatic potential.

The first term is the total ideal contribution

6 $$\beta F^{id}\left[\{N_{r}^{\left(c\right)}\}\right]=\sum_{r,c}\int dRN_{r}^{\left(c\right)}\left(R\right)\left(\ln\left[N_{r}^{\left(c\right)}\left(R\right)\right]-1\right)+\sum_{r,c}\int dRN_{r}^{\left(c\right)}\left(R\right)\Phi_{r}^{\left(b\right)}\left(R\right)+\sum_{i}\int drn_{i}\left(r\right)\psi_{i}^{0}\left(r\right)$$

The second term is the excess free energy, *F^ex^* [{*n*_*i*_(**r**)}], which only depends upon the monomer densities, *n*_+_(**r**) and *n*_–_(**r**), and includes excluded volume as well as mean-field Coulomb interactions. Here, it should be noted that monomers effectively exclude less volume than simple ions, even though they have identical diameters. Several ways to approximate this connectivity effect has been proposed, including thermodynamic perturbation theory (TPT1)^35–37^ and the Generalized Flory-Dimer theory (GFD).^38^ In Wertheim’s TPT framework, bond formation is mediated by directional attraction sites, and at the TPT1 level a chain monomer requires two singly bondable sites together with a closure relation suppressing ring formation. Our model takes a different route: rather than introducing explicit attraction sites, the association is governed by the stepwise equilibrium described above. Connectivity effects on excluded volume are treated through the simple approach utilized in an early version of polymer DFT,^39^ namely that the excluded volume by a monomer is effectively 80% as large as that by a simple ion, which in turn is approximated by the Carnahan–Starling equation of state.^40^ It should be noted that the precise way in which excluded volume is managed will not affect the conclusions of this work. The final term contains the chemical potential term given by

7 $$\beta \mu_{r,s}=\ln\left[\phi_{p}f\left(r,s\right)\right]+\beta \mu_{p}^{\mathrm{ex}}\left(r\right)$$

which consists of the ideal part ln­[ϕ_*p*_ *f*(*r*,**s**) ]. Here **s** is the vector of monomer types {*s*(*i*) = +1, −1: *i* = 1,*r*}. The excess term, μ_*p*_^ex^(*r*), is assumed to be independent of the charge of the polymer cluster in our theory, which is consistent with the commonly used Restricted Primitive Model, RPM. It will turn out (see below) that μ_*p*_^*ex*^(*r*) is a linear function of *r*.

Following the discussion above, the function *f*(*r*,*s*) assumes that the distribution of monomers in the polymer is determined by a constant *NN* bonding energy as well as *NN* Coulombic interactions. This gives

8 $$f\left(r,s\right)=Ke^{-\kappa_{++}N_{L}}e^{-\kappa_{+-}N_{U}}$$

where *N*_*L*_ and *N*_*U*_, are the number of like and unlike charged bonded monomers, respectively

9 $$N_{U}=\sum_{i=1}^{r-1}\left|s\left(i\right)-s\left(i+1\right)\right|/2$$

10 $$N_{L}=r-N_{U}-1$$

*K* is the normalization factor and κ_++_ and κ_+–_ determines the degree of association. We note that if we were to assume that Δ*G*_0_(++) = Δ*G*_0_(+−), then (κ_++_ – κ_+–_) /2 = γ would describe the *NN* Coulombic interaction, i.e., the difference in binding free energy between like and unlike nearest-neighbor pairs. As stated above, we do not invoke such a restriction on Δ*G*_0_, but we nevertheless only consider cases for which κ_++_ > κ_+–_.

In order to obtain *K*, we note that the number of monomers can also be written as *r* = ∑_*i* = 1_^*N*_*seg*_^ *n*_*i*_, where *N*_*seg*_ = *N*_*U*_ + 1 is the number of segments. Adjacent segments have opposite charge and their lengths are given by {*n*_1_, *n*_2_.··· *n*_*N*_*seg*__}.

13 K−1=∑Nseg=1∞⁡e−κ+−(Nseg−1)∏i=1Nseg∑ni=1∞⁡e−κ++(ni−1)(11)=∑Nseg=1∞e−κ+−(Nseg−1)(1−e−κ++)Nseg(12)=11−e−κ++−e−κ+−(13)

It is instructive to obtain an expression for the total density of charged species in the bulk solution, *n*_*b*_ = ϕ_*p*_⟨*r*⟩, where ⟨*r*⟩ is the average number of charged monomers per chain (we shall often use the more compact expression “chain length” for this quantity). Clearly the number of positive and negative monomers per chain will be equal on average. From the above expressions we obtain

16 nb=ϕpK∑Nseg=1∞⁡e−κ+−(Nseg−1)∏i=1Nseg∑ni=1∞⁡e−κ++(ni−1)∑k=1Nsegnk(14)=ϕpK∑Nseg=1∞⁡e−κ+−(Nseg−1)∏i=1Nseg∑ni=1∞⁡e−κ++(ni−1)Nseg∑n=1∞⁡e−κ++(n−1)n∑n=1∞⁡e−κ++(n−1)(15)=ϕp⟨Nseg⟩⟨n⟩(16)

where

17 $$\left\langle N_{\mathrm{seg}}\right\rangle =K\sum_{N_{\mathrm{seg}}=1}^{\infty }e^{-\kappa_{+-}\left(N_{\mathrm{seg}}-1\right)}{\left[\sum_{n=1}^{\infty }e^{-\kappa_{++}\left(n-1\right)}\right]}^{N_{\mathrm{seg}}}N_{\mathrm{seg}}$$

18 $$\left\langle n\right\rangle =\frac{\sum_{n=1}^{\infty }e^{-\kappa_{++}\left(n-1\right)}n}{\sum_{n=1}^{\infty }e^{-\kappa_{++}\left(n-1\right)}}$$

Thus, ⟨*N*_*seg*_⟩ is average number of segments per chain in the bulk and the average length of each segment is ⟨*n*⟩. The total density of charged monomers in the bulk can also be written as *n*_*b*_ = ϕ_*p*_⟨*r*⟩, where ⟨*r*⟩ = ⟨*N*_*seg*_⟩⟨*n*⟩. Now from (eq 13) we obtain

20 ∂⁡ln⁡K∂κ++=⟨Nseg⟩⟨n−1⟩(19)=e−κ++1−e−κ++−e−κ+−(20)

We can also write

22 ⟨Nseg⟩=∂⁡ln⁡K∂κ+−+1(21)=1−e−κ++1−e−κ++−e−κ+−(22)

These relations give

24 ⟨Nseg⟩⟨n⟩=11−e−κ++−e−κ+−(23)=K−1(24)

and thus, ⟨*n*⟩ =[1–e_^–κ^__^++^_]^−1^. The interplay between these parameters, across their full range, is illustrated in Figure 1. We have only investigated systems for which κ_++_ > κ_+–_ (except for the "toy model" where we allow their corresponding quanities to vary freely).

**Figure 1:**
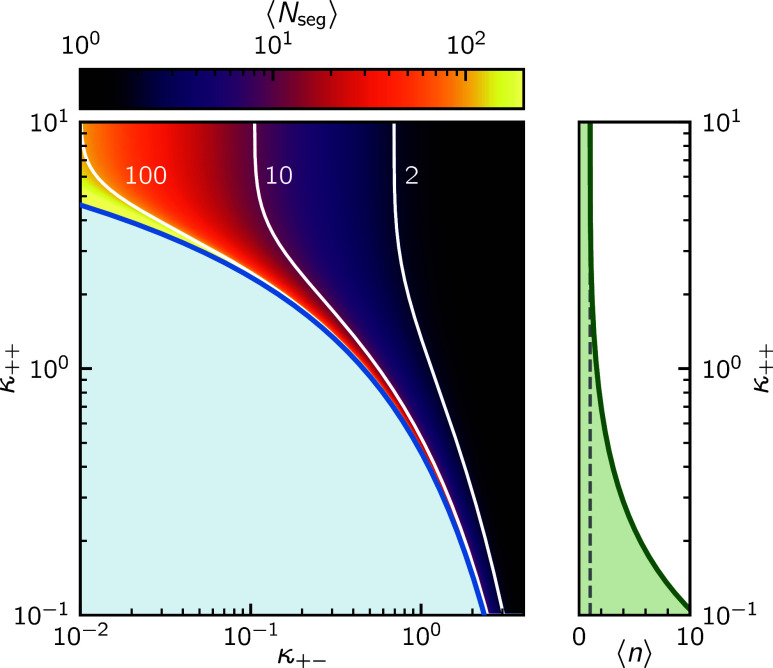
Visualization of the ⟨*N*_*seg*_⟩, ⟨*n*⟩ dependence on κ_++_ and κ_+–_. Regions with κ_++_ > κ_+–_ depict cases for which the backbone charges primarily (but not completely) *alternate (in the absence of external fields), while κ*_*+*–_ > κ_*++*_ *promotes the formation of block charges. The blue line and shaded area indicate the unphysical region, where 1/K diverges, or becomes negative.*.

The equilibrium polymer density is obtained by minimizing, Ω, which gives the following self-consistent expression for the polymer density

25 $$N_{r,s}\left(R\right)=\phi_{P}K\prod_{i=1}^{r}z_{s\left(i\right)}\left(r_{i}\right)\prod_{i=k}^{r-1}T\left(|r_{k}-r_{k+1}|\right)e^{-\kappa_{s\left(k\right)s\left(k+1\right)}}$$

Recall, *s*(*i*) = ± 1, signifies the valency of the *i*th monomer. The nonuniform fugacity term is given by

26 $$z_{s}\left(r\right)=e^{-\beta \psi_{s}\left(r\right)}$$

Furthermore, we have defined

27 $$\psi_{s}\left(r\right)=\frac{\delta F^{\mathrm{ex}}}{\delta n_{s}\left(r\right)}-\mu_{s}^{\mathrm{ex}}+\psi_{s}^{0}\left(r\right)$$

Thus, the potential ψ_*i*_(**r**) contains the electrostatic potential on the monomer of type *i* (= ± ), which includes the Donnan potential, as well as an excluded volume contribution. In addition, we have a contribution from any nonelectrostatic external potential. We also subtract an excess chemical potential term that is determined by the bulk conditions. Referring to (eq[Disp-formula eq7]), this implies that

28 $$\mu_{p}^{\mathrm{ex}}\left(r\right)=\sum_{i=1}^{r}\mu_{i}^{\mathrm{ex}}$$

As stated earlier, in our simplified theory we will find that μ_*i*_^*ex*^ will be independent of the monomer type *i*. Finally, we have

29 $$T\left(|r-r^{\prime }|\right)=\frac{e^{-\phi^{\left(b\right)}\left(|r-r\prime |\right)}}{\int dr^{\prime }e^{-\phi^{\left(b\right)}\left(|r-r\prime |\right)}}$$

We shall assume that monomers are bound at a fixed bond-length *b*, irrespective of their type, though the bond-energy will depend upon the pair of monomers involved, i.e.

30 $$T\left(\left|r-r^{\prime }\right|\right)=\frac{\delta \left(\left|r-r^{\prime }\right|-b\right)}{4\pi b^{2}}$$

It is convenient to define the following expressions

$$\begin{array}{l} g_{s}\left(r_{i}\right)=e^{-\beta \psi_{s}\left(r\right)/2}\\ S_{\mathrm{ss}\prime }\left(r,r^{\prime }\right)=g_{s}\left(r\right)T\left(|r-r^{\prime }|\right)g_{s\prime }\left(r^{\prime }\right)\\ F_{n}^{\left(s\right)}\left(r_{1}\cdot \cdot \cdot r_{n+1}\right)=\prod_{i=1}^{n}S_{\mathrm{ss}}\left(r_{i},r_{i+1}\right)\\ F_{0}^{\left(s\right)}\left(r_{1}\right)=g_{s}\left(r_{1}\right) \end{array}$$

The last two terms corresponds to an *n*-segment distribution (unnormalized) of a single type of charged monomers (type *s*). We now note that the vector {*s*(1), *s*(2), ···*s*(*r*)} appearing in (eq 18) can be grouped into *n*_*i*_-segments of like charged monomers, i.e., *N*_*seg*_ segments of type {*s*_*i*_ = ± 1: *i* = 1, *N*_*seg*_} with lengths given by {*n*_*i*_: *i* = 1, *N*_*seg*_}. Note that the, *s*_*i*_, will alternate between +1 and −1, and the sum of all the *n*_*i*_ is equal to *r*. The total ensemble of chains is obtained by summing over the number of segments, *N*_*seg*_, and the number of (like-charged) monomers within each segment, *n*_*i*_. We reiterate that the segments will alternate in charge, but for *N*_*seg*_ odd, we will have the end segments be either both positive or both negative and, for *N*_*seg*_ even, the end segments will be of opposite charge.

Suppose for the moment we consider the subset of chains with a fixed number of segments, *N*_*seg*_, but otherwise all possible number, *n*_*i*_, of like-charged monomers within each segment. Let us calculate the total positive charge density at some position, **r** for such chains. We denote this density as, *n*_*N*_*seg*__^(+)^. We begin by defining the following contracted (unnormalized) segment density

31 $${\mathrm{F̅}}_{n}^{\left(s\right)}\left(r,r^{\prime }\right)=\int dr_{1}\cdot \cdot \cdot dr_{n}\delta \left(r_{1}-r\right)\delta \left(r_{n}-r^{\prime }\right)\prod_{i=1}^{n-1}S_{\mathrm{ss}}\left(r_{i},r_{i+1}\right)$$

We then sum the contracted distributions over all possible numbers of like-charged monomers within each segment, appropriately weighted by the *NN* Coulombic penalty function

32 $${\mathrm{F̂}}^{\left(s\right)}\left(r,r^{\prime }\right)=\sum_{n=0}^{\infty }e^{-\kappa_{++}n}{\mathrm{F̅}}_{n}^{\left(s\right)}\left(r,r^{\prime }\right)$$

These *F̂* ^(*s*)^(**r**, **r**′) are then linked using *S*_+–_(**r**, **r**′) to form a chain of *N* + 1 segments (with alternating charge) and terminating in a positive segment. As discussed above these are constructed such that odd and even values *N* + 1 will have constraints on the charge of the terminal segments. Below, are the examples *N* = 0, 1, 2 and 3

$$\begin{array}{l} g_{+}\left(r_{1}\right){\mathrm{F̂}}^{\left(+\right)}\left(r_{1},r_{2}\right)g_{-}\left(r_{1}\right){\mathrm{F̂}}^{\left(-\right)}\left(r_{1},r_{2}\right)S_{-+}\left(r_{2},r_{3}\right){\mathrm{F̂}}^{\left(+\right)}\left(r_{3},r_{4}\right)\\ g_{+}\left(r_{1}\right){\mathrm{F̂}}^{\left(+\right)}\left(r_{1},r_{2}\right)S_{+-}\left(r_{2},r_{3}\right){\mathrm{F̂}}^{\left(-\right)}\left(r_{3},r_{4}\right)S_{-+}\left(r_{4},r_{5}\right){\mathrm{F̂}}^{\left(+\right)}\left(r_{5},r_{6}\right)\\ g_{-}\left(r_{1}\right){\mathrm{F̂}}^{\left(-\right)}\left(r_{1},r_{2}\right)S_{-+}\left(r_{2},r_{3}\right){\mathrm{F̂}}^{\left(+\right)}\left(r_{3},r_{4}\right)S_{+-}\left(r_{4},r_{5}\right){\mathrm{F̂}}^{\left(-\right)}\left(r_{5},r_{6}\right)S_{-+}\left(r_{6},r_{7}\right){\mathrm{F̂}}^{\left(+\right)}\left(r_{7},r_{8}\right) \end{array}$$

We will denote these functions as *c*_*N*_^(+)^(**r**_1_, ···**r**_2*N*+2_). This function can be contracted as follows

33 $${\mathrm{c̅}}_{N}^{\left(+\right)}\left(r\right)=\int dr_{1}\cdot \cdot \cdot dr_{2N+2}\delta \left(r_{2N+2}-r\right)c_{N}^{\left(+\right)}\left(r_{1},\cdot \cdot \cdot r_{2N+2}\right)$$

The total positive density charge contribution due to chains of this type (fixed *N*_*seg*_) is given by

34 $$n_{N_{\mathrm{seg}}}^{\left(+\right)}\left(r\right)=\phi_{P}Ke^{-\kappa_{+-}\left(N_{\mathrm{seg}}-1\right)}\sum_{N=1}^{N_{\mathrm{seg}}}{\mathrm{c̅}}_{N_{\mathrm{seg}}-N}^{\left(+\right)}\left(r\right){\mathrm{c̅}}_{N-1}^{\left(+\right)}\left(r\right)$$

Finally, we can sum this over *N*_*seg*_ to obtain

35 $$n^{\left(+\right)}\left(r\right)=\phi_{P}K\sum_{N_{\mathrm{seg}}=1}^{\infty }e^{-\kappa_{+-}\left(N_{\mathrm{seg}}-1\right)}\sum_{N=1}^{N_{\mathrm{seg}}}{\mathrm{c̅}}_{N_{\mathrm{seg}}-N}^{\left(+\right)}\left(r\right){\mathrm{c̅}}_{N-1}^{\left(+\right)}\left(r\right)$$

which can be rewritten as

36 $$n^{\left(+\right)}\left(r\right)=\phi_{P}K{ĉ}^{\left(+\right)}\left(r\right){ĉ}^{\left(+\right)}\left(r\right)$$

37 $${ĉ}^{\left(+\right)}\left(r\right)=\sum_{N_{\mathrm{seg}}=1}^{\infty }e^{-\kappa_{+-}N_{\mathrm{seg}}}{\mathrm{c̅}}_{N_{\mathrm{seg}}}^{\left(+\right)}\left(r\right)$$

Similarly, we obtain for negative charges

38 $$n^{\left(-\right)}\left(r\right)=\phi_{P}K{ĉ}^{\left(-\right)}\left(r\right){ĉ}^{\left(-\right)}\left(r\right)$$

We can obtain the following recursion formulas for *ĉ* ^(+)^(**r**) and *ĉ* ^(−)^(**r**).

39 $${ĉ}^{\left(+\right)}\left(r\right)=g_{+}\left(r\right)+e^{-\kappa_{+-}}S_{+-}\left(r,r^{\prime }\right)*{ĉ}^{\left(-\right)}\left(r^{\prime }\right)+e^{-\kappa_{++}}S_{++}\left(r,r^{\prime }\right)*{ĉ}^{\left(+\right)}\left(r^{\prime }\right)$$

40 $${ĉ}^{\left(-\right)}\left(r\right)=g_{-}\left(r\right)+e^{-\kappa_{+-}}S_{+-}\left(r,r^{\prime }\right)*{ĉ}^{\left(+\right)}\left(r^{\prime }\right)+e^{-\kappa_{--}}S_{--}\left(r,r^{\prime }\right)*{ĉ}^{\left(-\right)}\left(r^{\prime }\right)$$

with the asterix defining a convolution. The above formulas then form a set of self-consistent equations to be solved for the overall densities.

### Interactions between Charged Surfaces, in the Presence of Dissociated 1:1 Salt

We will explore interactions between charged surfaces immersed in an aqueous solution containing a monovalent (1:1) salt, under the assumption that a fraction of the simple ions bond to form ion clusters. The latter category of ions, which we denote as “monomers”, follow the cluster formalism described above, whereas the nonclustering part remain as “dissociated” ions. With this distinction, the grand potential, eq 5 needs to be modified somewhat, to include the corresponding free energies and chemical potentials of the dissociated ions. In particular, the excess free energy *F* ^*ex*^ is now a function of monomer as well as dissociated ion densities. This function can in turn be decomposed into a term that describes Coulomb interactions between all charged species (including wall charges), and another term that accounts for excluded volume interactions. The excluded volume free energy cost is approximated by a Carnahan–Starling expression, where all particles carry a hard sphere of diameter *d_hs_* = 3 Å, but with the monomer excluded volume reduced by a factor, due to overlapping of free volume between adjacently bonded monomers. This is a similar approximation to that used in an earlier study.^39^ We emphasize that the conclusions made in the current work is not sensitive to the way in which excluded volume effects are approximated.

We recall that monomers are characterized by the common notation “*s*”, the sign of which identifies the monomer valency. We shall, in a similar fashion, use “*d*” to characterize dissociated (nonclustering) ions, where again the sign identifies the valency of those ions (*d* should not be confused with *d*_*hs*_).

The final term in eq 20 can be split into two separate contributions

41 $$\psi_{s}^{0}\left(r\right)=V_{\mathrm{wall}}\left(r\right)+\mathrm{se}\psi_{s}^{D_{s}}\left(r\right)$$

where the first term describes the nonelectrostatic component of the surface potential. The two walls are assumed to be parallel, with an infinite extension along the (*x*,*y*) coordinates, and located at *z* = 0 and *z* = *h*, respectively. Thus, *V*_wall_ is zero in the bulk, as well as in the region *d*_*hs*_/2 < *z* < (*h* – *d*_*hs*_/2) and infinite elsewhere. The last term describes the monomer valency (*s*) multiplying the elementary charge (*e*) times a constraining potential, ψ_*s*_^*D*_*s*_^(**r**), which is zero in the bulk solution and equal to the Donnan potential, ψ^Donnan^, inside the slit, for systems at full electrochemical equilibrium. However, we will also explore the option of a “semi-restricted” equilibrium. In this case we will assume that the surface charge is neutralized only by the dissociated ions, while the total charge of monomers, summed over all the ionic clusters, is assumed to be zero. The reasons for this latter assumption is argued below.

The corresponding expression to (eq 34) for dissociated ions reads

42 $$\psi_{d}^{0}\left(r\right)=V_{\mathrm{wall}}\left(r\right)+\mathrm{de}\psi_{d}^{D_{d}}\left(r\right)$$

where we recall that *d* is the valency of the dissociated charges. It should be noted that ψ_*d*_^*D*_*d*_^(**r**) also acts on the wall charges. At full equilibrium, ψ_*d*_^*D*_*d*_^(**r**) is equal to the Donnan potential, for all species inside the slit. At semirestricted equilibrium, where the surface charge is fully neutralized by dissociated ions, the potential is slightly different, as we shall demonstrate below. All calculations are made at room temperature, with a Bjerrum length of 7.16 Å. The bond length between connected monomer is set to *b* = 4 Å.

Defining *g*_*s*_(*h*) ≡ Ω_eq_/*A*–*p*_*bh*_, where *p*_*b*_ is the bulk pressure, we can write the *net* interaction free energy as Δ*g*_*s*_ = *g*_*s*_(*h*) – *g*_*s*_(*h*_max_), where *h*_max_ is at large separation so that the net interaction can be assumed to be zero. The net pressure acting perpendicularly to the flat surfaces is denoted *p*_net_, with *p*_net_ = −∂*g*_*s*_/∂*h*. This quantity can be evaluated either analytically or discretely (from *g*_*s*_(*h*)) and the agreement between these forms an important and useful check of the cDFT codes.

## Results

While most of our efforts will be devoted to models of ion clusters (with charged monomers) it is instructive to first probe the qualitative responses to parameter changes of toy *A*/*B* mixture model, composed of simple uncharged hard-sphere monomers, labeled *A* and *B*.

### Toy Model

Here, the *A* and *B* particles are simple hard spheres, of the same diameter: *d* = 3 Å. They are only distinguishable by their affinity to the surfaces. Both monomer types are excluded from the internal surface region by *V*_wall_. However, the *A* monomers also experience an adsorption potential *w*, which at the left wall can be written as

43 $$w\left(z\right)=\{\begin{array}{ll} 0; & z\geq z_{c}\\ C\left({\left(z-d/2\right)}^{2}-{\left(z_{c}-d/2\right)}^{2}\right)/d^{2} & z<z_{c} \end{array}$$

where we have set *z*_*c*_ = 2*d* and β*C* = 0.45. The total affinity between an *A* monomer and the surfaces separated by *h* is *W*_wall_(*h*, *z*) = *w*(*z*) + *w*(*h* – *z*). For clarity we will replace the κ_++_ and κ_+–_ notation with κ_*AA*_ and κ_*AB*_ in the toy model. It should be noted that in our *A*/*B* mixture, there is no constraint on the sign of γ, in contrast to ion clusters, where we expect oppositely charged monomers to be bonded more tightly on account of the extra Coulomb attraction.

The resulting surface interactions and monomer density profiles are displayed in Figure 2. The average polymer length is held fixed at ⟨*r*⟩ ≈ 101 but the degree of “polarization” as determined by the *bulk* parameter, ⟨*n*⟩, is varied. A large value of κ_*AA*_ results in chains with predominately an alternating sequence (*ABAB*···). A value of κ_*AA*_ = 10 is high enough to maintain this alternating sequence even in the presence of surfaces attractive to A. The corresponding surface interaction (graph **a**) is rather short-ranged and monotonically attractive, due primarily to bridging attractions. For adsorbing chains one may expect an intermediate barrier, as surfaces approach each other and adsorbed chains first encounter each other’s excluded volume. This barrier is present in monodisperse chains but is absent in adsorbed “living” chains, as described in the current model.^41^ Living chains are able to alleviate hard core repulsions by suitably varying their length. By decreasing κ_*AA*_ we are able to induce polarization in the chains, but it is only for values significantly below 1 that we observe a strong effect on the surface forces. Nevertheless, at κ_*AA*_ = 0.1, the range of the attraction has increased somewhat, and κ_*AA*_ = 0.01 leads to a very long-ranged attractive tail. Note that the latter value effectively generates (polydisperse) homopolymers.

**Figure 2:**
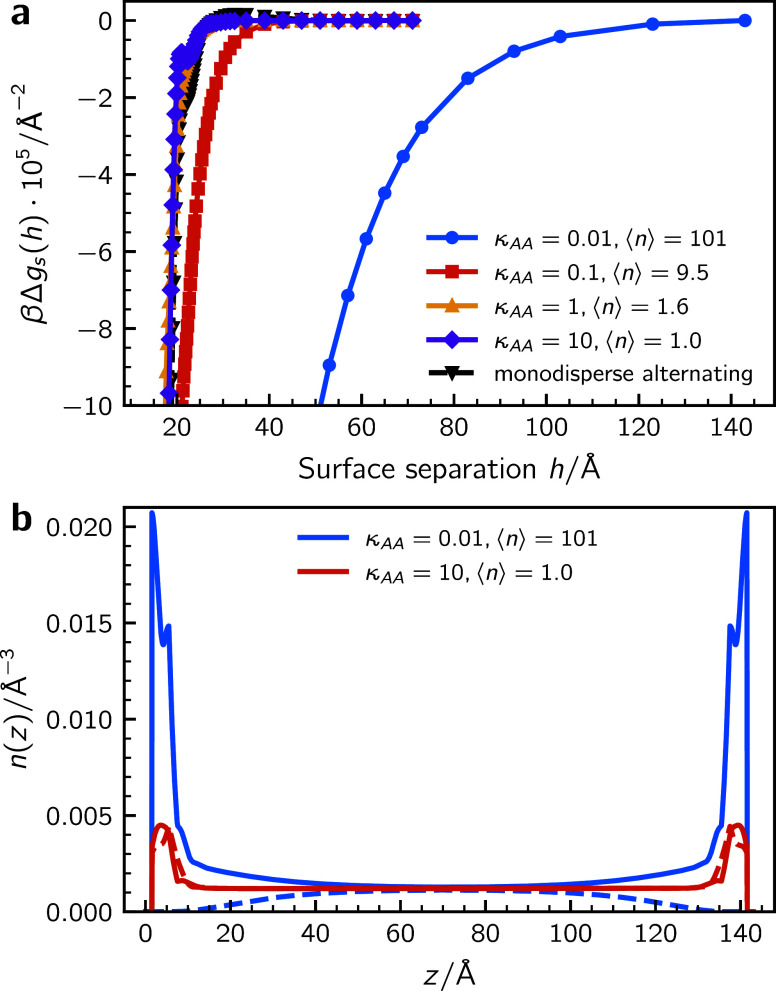
(**a**) *A*/*B* mixtures: net interaction free energies. (**b**) *A*/*B* mixtures: density profiles. Solid and dashed lines display distributions of *A* and *B* monomers.

The density profiles in Figure 2(**b**), show that a decrease in κ_*AA*_ causes a much stronger adsorption at the walls. In contrast, with an alternating sequence (large κ_*AA*_), the required presence of nonadsorbing *B* effectively reduces the overall surface affinity.

### Ion Clusters

We now turn our attention to ion clustering, and the influence of these clusters on interactions between charged surfaces. In earlier work, we have shown that, if a *dominant* fraction of ions form largely neutral (or at most singly charged) and monodisperse clusters, then quite strong and long-ranged interactions are produced.^23,24^ One could argue that significant clustering is not consistent with conductivity measurements. While there is a tendency for the (linear) dependence of conductivity on concentration to decrease at high ionic strengths, the effect is rather modest, at least for *NaCl*_(*aq*)_ solutions.^42^

Given these arguments, we will here consider a rather different scenario to what we did previously. More specifically, we will consider here cases where only a *minor* fraction of the ions form clusters of the *polydisperse* type described in the present model. We begin by solving the cDFT, as is, assuming so-called *full electrochemical equilibrium* for all the charged species.

#### Full Equilibrium

For full equilibrium, we have that the electrochemical equilibria both associated (monomers) and dissociated ions give rise identical Donnan potentials, i.e., ψ_*s*_^*D*_*s*_^ = ψ_*d*_^*D*_*d*_^ = ψ^Donnan^. In this case, the impact from ion clusters on surface interactions is quite modest. This is shown in Figure 3 for an ion mixture of 1 M dissociated electrolyte, in the absence and presence of 20 mM of “monomer salt”. The latter ions are characterized by an average bulk polymer length of ⟨*r*⟩ = 25 and average like-charged segment length of ⟨*n*⟩ ≈ 1.14. Thus, there are significantly more dissociated than associated ions and the degree of polarization allowed for the clusters is quite low, as ⟨*n*⟩ is close to unity. The surface affinity is much greater for dissociated ions than ion clusters (polyampholytes). The reason is that the net charge of the polyampholytes typically speaking is quite modest (close to zero) leaving clusters depleted from the near-surface region and having very little impact on the surface forces.

**Figure 3:**
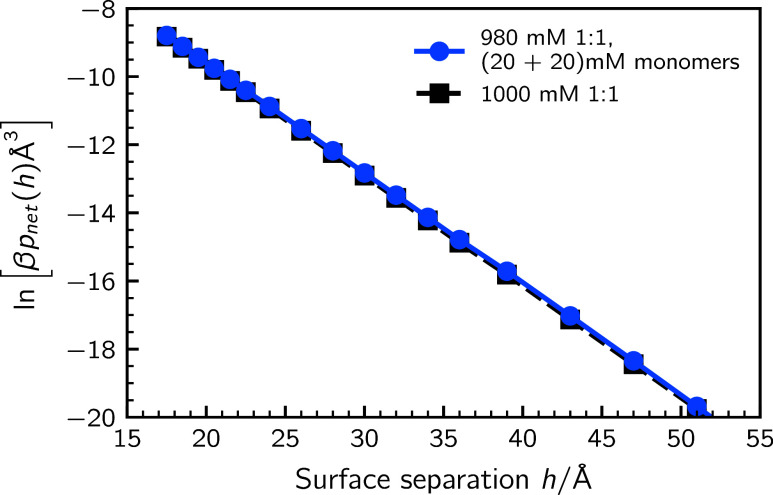
A comparison between the long-ranged net pressure tails at approximately 1 M dissociated salt, in the absence and presence of 20 mM “monomer salt”. The latter ion type can polymerize as well as polarize: ⟨*r*⟩ = 25 and ⟨*n*⟩ ≈ 1.14. While not shown here, the results are very similar to fully alternating chains, ⟨*n*⟩ = 1.00.

#### Semirestricted Equilibrium

In the cDFT as formulated here, the propensity to form ion clusters is determined by nearest-neighbor parameters κ_+–_ and κ_++_. These parameters in essence manifest the intrinsic free energy of the clusters (albeit in the bulk fluid). Unfortunately, the model contains no further considerations of the internal correlations within the clusters, which will likely be very strong, considering the local dielectric constant is diminished due to the lack of freely rotating water molecules within. These internal thermodynamic considerations will be nonlocal, i.e., they will act beyond nearest-neighbors. A plausible outcome of this is that the accumulation of charge on some part of a given cluster will be balanced to some degree by a counter charge within that cluster and in adjacent, neighboring clusters. This assumes that inter- and intracluster correlations are stronger than those involving dissociated ions. For this reason, it is plausible to impose a constraint on the solutions of the cDFT, which reflects the different correlations between monomer ions within clusters and those among dissociated ions.

A simple way to realize these differences is applied here. Specifically, we will make the assumption that the neutralization of the charged surfaces is carried out by the dissociated ions, while the charge of the clustered (monomer) ions within the region between the surfaces sums to zero. This allows for charge polarization of the clusters, but insists that the clusters (due to their stronger correlations) must essentially neutralize themselves and/or each other. This global constraint can be viewed as one of the simplest examples of a potential family of cluster constraints that reflect the different responses of bound and dissociated ions. In this context, we can also mention that the general dynamics of clusters are expected to be somewhat slower than dissociated ions, including the rearrangement of ions within clusters. This has often been used as an argument to impose a semirestricted equilibrium constraint on cluster response. It should be noted that a “cluster” actually consists of a “core” part plus an atmosphere of associated ions on the surface of the core. The ions in that atmosphere are assumed to be counted as part of the “free ions” and the cores are what we treat as polymers in our model. Core ions do not easily dissociate/associate within the time frame of surface force experiments. However, cores can polarize through ion diffusion (± swapping) within a core, or else exchanging charge with other cores with which they collide. Moreover, as we shall demonstrate below, the clusters tend to be depleted from the surfaces anyway, presumably due to a higher surface charge density of simple ions. This means that the restriction we impose only have a minor impact on the density distributions, but remarkably enough still affects the surface interactions considerably. While neither of these arguments constitute a “proof”, we argue that it is of interest to explore how a minor deviation from complete chemical equilibrium may influence surface forces. The presence of such deviations is perhaps the origin behind some rather deviating results reported by various AFM/SFA investigations in this field. In fact, there is a recent publication that at least to some extent supports this hypothesis.^43^ In those SFA experiments, they find surface forces that depend on the speed at which the surfaces are brought together (or are separated). While they studied interactions across ionic liquids, the findings are possibly general enough to apply also to aqueous salt solutions, although this remains to be verified.

Pragmatically, this constraint is carried out in the cDFT by allowing different values for ψ_*s*_^*D*_*s*_^ and ψ_*d*_^*D*_*d*_^. The consequences are rather dramatic. As can be seen in Figure 4, the slight deviation from complete equilibrium afforded by this constraint, generates a long-ranged repulsive force, even with a minor fraction of clustering species. This is the same system as displayed in Figure 3, at full equilibrium. The interactions at full and semirestricted equilibrium agree quite well at short separations, but deviate dramatically at intermediate and large separations.

**Figure 4:**
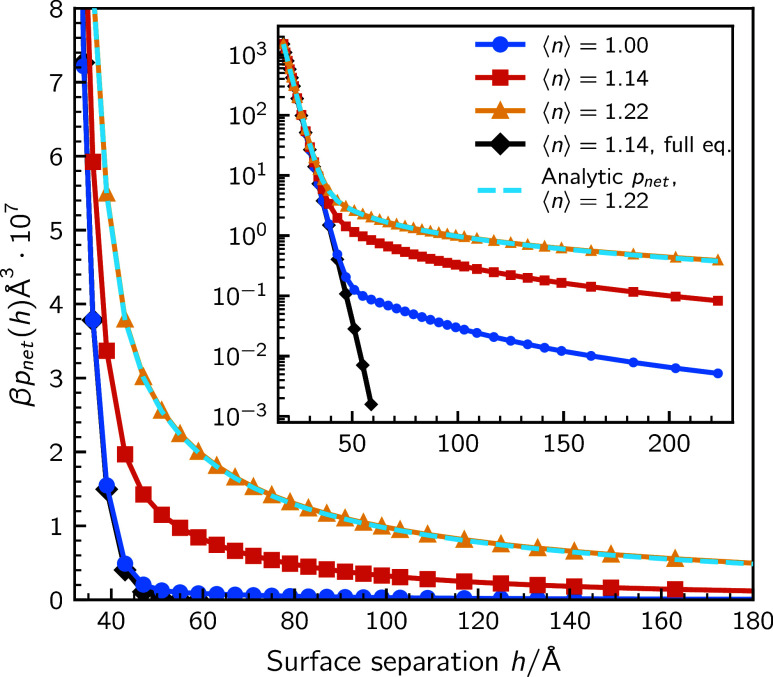
Net pressures, at semirestricted equilibrium, with ⟨*r*⟩ = 25 and various degrees of polarizability, ⟨*n*⟩. The bulk solution contains 980 mM dissociated salt and 20 + 20 mM monomers (cat- and anionic). The inset displays the long-range decay, as illustrated by the logarithm of the net pressure. The dashed line, for ⟨*n* ⟩ = 1.22, demonstrates the equivalence between the analytic (*p*_net_ = −∂*g*_*s*_/∂*h*) and discrete (*p*_net_ = −δ*g*_*s*_/δ*h*) free energy derivatives. The corresponding net pressure at full equilibrium is shown for reference, by a green line with diamonds.

One of the most peculiar aspects of “anomalous underscreening” is the way in which the interactions between charged surfaces displays a range that either is roughly constant, or even *increases* as more salt is added, beyond concentrations of 1 M, or thereabouts. Let us explore predictions in this regime of the semirestricted equilibrium constraint hypothesis.

We first imagine a salt solution of 1 M, in which some small fraction are cluster-forming ions (monomers). Upon an increase of the overall concentration to 2 M, one would anticipate that the fraction of clustering ions increases, though the *specific* molecular mechanism underlying the cluster formation is beyond the scope of this work. We examine possible scenarios, given these preliminary considerations. In Figure 5, we have plotted interaction free energies (graph (**a**)) and net pressures (graph (**b**)) at an overall total salt concentration of 1 and 2 M, where we make the qualitative assumption that a smaller fraction of ions cluster at the lower concentration. We see that if this doubling of the overall salt concentration leads to a 10-fold increase of the clustering ion (monomer) concentration (say 20 mM ⇒ 200 mM) then the surface forces remain similar. But if the cluster tendency grows more rapidly with ionic strength (say 1 mM ⇒ 200 mM), then we find a substantially increased strength at the higher concentration, although the range appears to be roughly constant.

**Figure 5:**
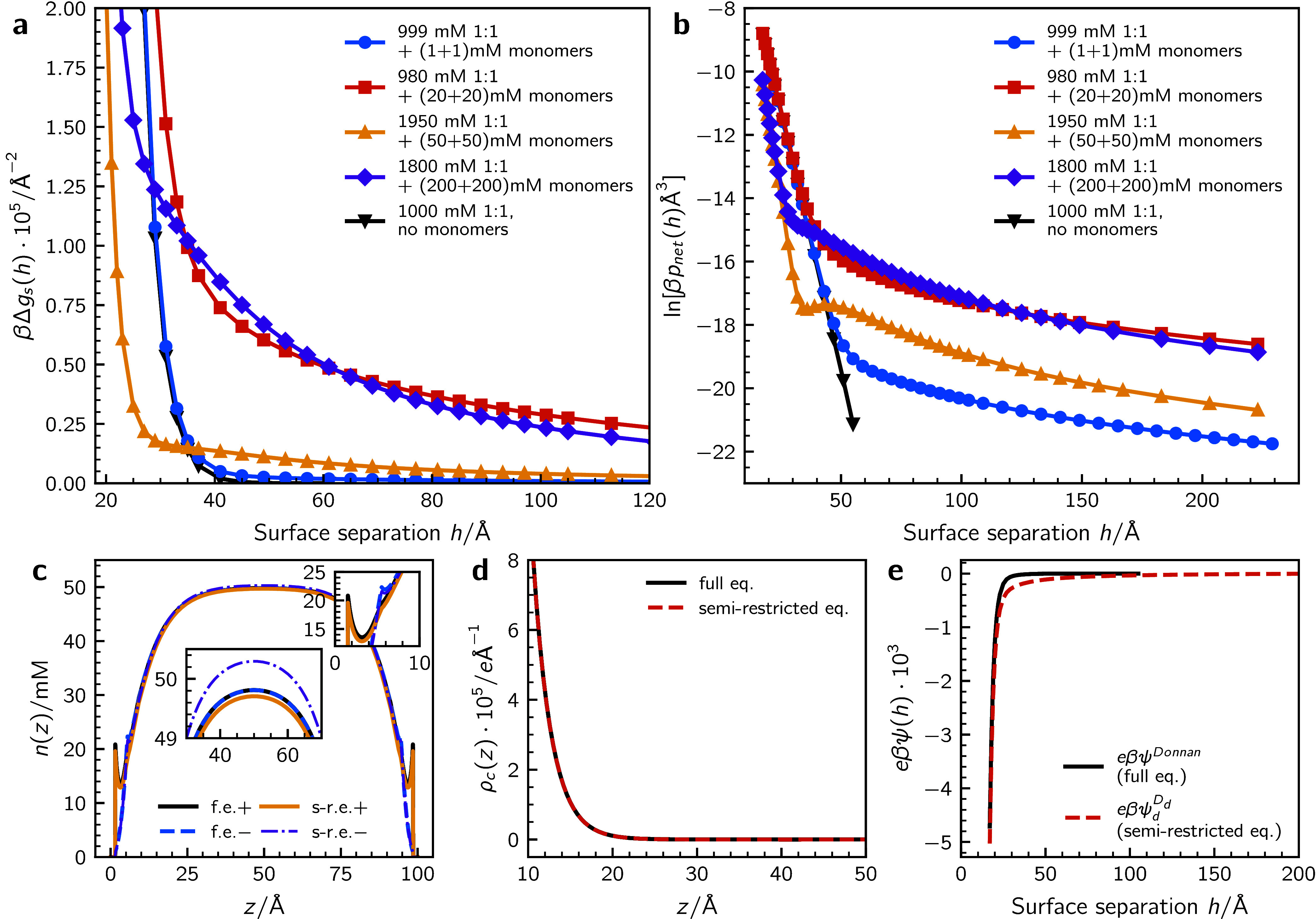
(**a**) Illustrations of possible effects on surface interactions, obtained by adding salt, under the assumption of semiequilibrium conditions and concentration-induced clustering. Bond parameters (κ_++_ and κ_+–_) are chosen such that ⟨*r*⟩ = 25 and ⟨*n*⟩ ≈ 1.14 in all cases. (**b**) Long-range *p*_net_ decay. (**c**) Comparing density (*n*(*z*)) and (**d**) charge density (ρ­(*z*)) profiles at a surface separation of 100 Å. The dashed lines in graph (**c**) show anion profiles. The bulk concentration of monomers and dissociated salt is 50 mM and 1950 mM (commensurate with a 2 M salt solution, 50 mM of which form clusters). Results are shown from calculations at full (f.e.) and semirestricted (s-r.e.) equilibrium. (**e**) Comparing the variation of restriction potentials ψ^Donnan^ (full equilibrium) and ψ_*d*_^*D*_*d*_^ (semirestricted equilibrium) with separation. The bulk concentrations of monomers and dissociated salt are 50 mM and 1950 mM, respectively.

The origin of the strong effect that this seemingly modest deviation from full equilibrium can be sought as follows. In Figure 5(**c**),(**d**), we compare monomer and charge density profiles at a surface separation of 100 Å, under full and semirestricted equilibrium, in a solution containing 1950 mM dissociated ions, and (50 + 50) mM cat- and anionic monomers. The depleted monomer profiles that we discussed earlier, are explicitly shown in graph (**c**), where we also note a strong similarity between profiles established at full and semirestricted equilibrium. Nevertheless, there is a small but detectable (inset) difference that progresses all the way to the midplane of the slit. On the other hand, graph (**d**) illustrates that the corresponding *charge density* profiles are essentially identical. This in turn implies an almost identical local potential variation across the slit.

In Figure 5(**e**) we see that the strength of the constraining potential ψ_*d*_^*D*_*d*_^ decays much slower than its correspondence at full equilibrium, ψ^Donnan^. The deviation is rather small in absolute terms. Nevertheless, this pinpoints the origin of the large differences we have seen between surface interactions at full and semirestricted equilibrium.

Finally we consider the effect of cluster size, i.e., average polymer length. Our two-parameter description of ion–ion bonds, suggests that there is more than one way to adjust our parameters in order to change the average degree of polymerization, ⟨*r*⟩—cf. Figure 1. In Figure 6 we show examples of how the surface forces can change, at semirestricted equilibrium, if the average polymer length is reduced. We note, perhaps somewhat surprisingly, that the repulsion might become stronger as the average cluster size drops. We should then note that this reduction is accompanied by an overall increase of the cluster concentration, since the density of the cluster-forming monomers is constant. Another aspect to keep in mind is that for very small average cluster sizes, the underlying mechanisms that (might) support semirestricted conditions become weaker, i.e., for tiny average cluster sizes, there is a smaller difference in terms of charge density, when compared with fully dissociated ions.

**Figure 6:**
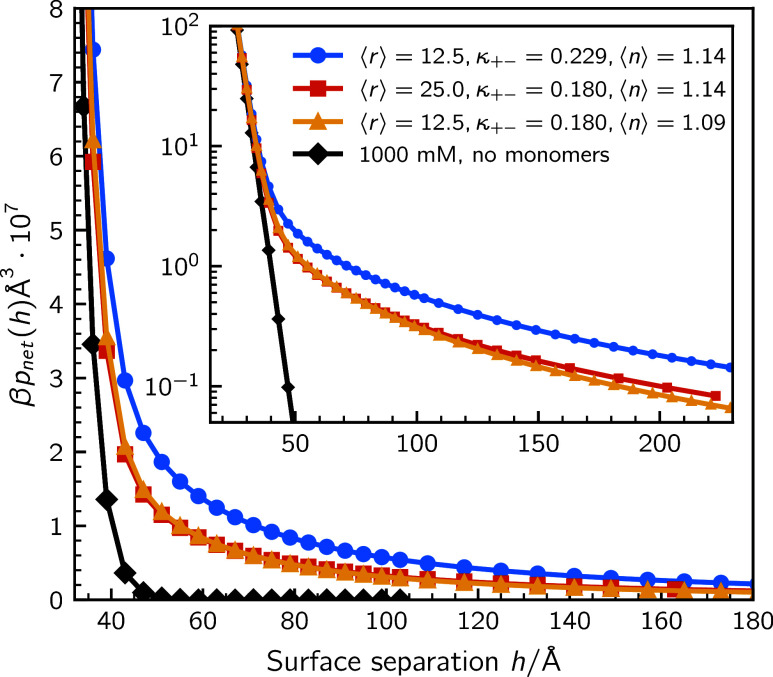
Examples of how the surface interactions can vary with the average cluster size, under the assumption of semirestricted equilibrium. In the cluster-containing solutions, the monomer concentration is (20 + 20) mM and the concentration of dissociated salt is 980 mM. The insert presents the long-ranged net pressure decays.

## Conclusions

We have developed a cDFT formalism to treat mixtures of simple *A* and *B* particles, that can reversibly associate by breaking or forming *A*–*A*, *A*–*B* and *B*–*B* bonds. While the cDFT treatment is general, we have devoted particular attention to its application on a hypothetical scenario in which simple monovalent ions cluster together by forming reversible bonds at high salt concentrations in aqueous solutions. A similar scenario could appear in ionic liquids, or ionic liquid + solvent mixtures, with an analogous cluster formation mechanism. We have not investigated what underlying molecular mechanisms might generate the reversible bonds in this work.

Assuming that only a rather small fraction of the total amount of ions polymerize to form (widely polydisperse) linear clusters, our mean-field calculations indicate that the presence of these clusters only have a minor influence on the interaction between charged surfaces, at complete equilibrium. We note that dissociated ions, with a much higher concentration and surface charge density than a typical cluster, accumulate at the surfaces, and displace the clusters so that the latter are depleted in these regions.

The expected strong correlations within and between clusters, perhaps combined with an expected slow cluster dynamics suggests a simple semiconstrained model that surface neutralization is entirely accomplished by dissociated ions. This raises a more general question as to what extent a concentrated ionic solution necessarily maintains complete equilibrium during a typical SFA experiment. We have explored a semirestricted model and found that only a very small fraction of cluster-forming ions suffices to generate strong and long-ranged surface forces. We speculate here that this phenomenon may be related to the observed “anomalous underscreening” though further investigations on other types of constrained systems are clearly required to draw firmer conclusions. It is possible that the constraint used here exaggerates the differences in cluster and dissociated ion correlations. In “real” solutions one might anticipate that deviations from complete equilibrium primarily is relevant for rather large clusters. Still, we believe that our approach is a first step in establishing the use of plausible constraints in simple theories (such as the mean-field cDFT) to investigate these phenomena. We find it particularly intriguing that even though the semiequilibrated restriction has a very small influence ion distributions, and a vanishing impact on charge density distributions, it nevertheless produces quite dramatic changes to the surface forces, also when the total cluster concentration is quite low. This type of generalized cDFT formalism could also prove useful to a broader range of systems than that investigated in this work.

## Data Availability

The cDFT code is available at Zenodo: 10.5281/zenodo.18619972.
